# The Prevalence of Enteropathy Symptoms from the Lower Gastrointestinal Tract and the Evaluation of Anorectal Function in Diabetes Mellitus Patients

**DOI:** 10.3390/jcm10030415

**Published:** 2021-01-22

**Authors:** Małgorzata Reszczyńska, Radosław Kempiński

**Affiliations:** 1 Department of Gastroenterology and Hepatology, Wrocław University Hospital, 50-556 Wrocław, Poland; 2 Department of Gastroenterology and Hepatology, Wrocław Medical University, 50-367 Wrocław, Poland; radoslaw.kempinski@umed.wroc.pl

**Keywords:** diabetes mellitus, enteropathy, high resolution anorectal manometry, neurological disorders

## Abstract

Complications affecting the gastrointestinal tract often occur in the course of diabetes mellitus (DM). The aim of this study was to evaluate enteropathy symptoms and anorectal function using high-resolution anorectal manometry (HRAM). Fifty DM patients and 20 non-DM controls were enrolled into the study. Clinical data and laboratory tests were collected, physical examination and HRAM were performed. Symptoms in the lower gastrointestinal tract were reported by 72% of patients. DM patients with a long disease duration reported anal region discomfort (*p* = 0.028) and a sensation of incomplete evacuation (*p* = 0.036) more often than patients with shorter diabetes duration. Overall, DM patients had a lower maximal squeeze pressure (MSP) (*p* = 0.001) and a higher mean threshold of minimal rectal sensation (*p* < 0.01) than control subjects. They presented with enhanced features of dyssynergic defection than the control group. MSP and maximal resting pressure (MRP) were significantly lower in the group of long-term diabetes (*p* = 0.024; *p* = 0.026 respectively) than in patients with a short-term diabetes. The same observation was noted for patients with enteropathy symptoms that control for MSP (*p* < 0.01; *p* < 0.01; *p* = 0.03) and MRP (*p* < 0.001; *p* = 0.0036; *p* = 0.0046), respectively, for incontinence, constipation, and diarrhea. Symptoms in the lower gastrointestinal tract are often reported by DM patients. All DM patients have impaired function of the external anal sphincter and present enhanced features of dyssynergic defecation and also impaired visceral sensation. Patients with long-standing DM and patients with enteropathy symptoms have severely impaired function of both anal sphincters.

## 1. Introduction

The prevalence of diabetes mellitus is increasing rapidly due to the worldwide obesity epidemic. Complications occurring over the course of this disease can affect many organs including the gastrointestinal tract [1]. Diabetic enteropathy is a relevant complication, which has a multi-factorial and complex etiology. According to the latest data, many different mechanisms of action lead to the dysfunction of the enteric nervous system, such as microangiopathy, autonomic neuropathy, myopathy, polyneuropathy, and gut microbiome disturbances, which cause dysmotility in the gastrointestinal tract [2,3]. Many factors including hyperglycemia, oxidative stress, and neuro-inflammatory processes reduce levels of nerve growth factors and induce intracellular changes in neurons [2]. Data concerning abnormalities in the upper gastrointestinal tract are published more often than data containing abnormalities of the lower gastrointestinal tract [4]. Complaints such as chronic constipation, anal regional pain, chronic diarrhea, and incontinence are important but often overlooked issues in interdisciplinary medical care of diabetic patients. Anorectal manometry is a routinely used diagnostic method in proctological practice, whereas high-resolution anorectal manometry (HRAM) is a relatively new diagnostic method. It provides detailed data according to anorectal function, which can be abnormal in diabetes mellitus (DM) [5]. According to Eldosky et al., diabetic patients with neuropathy and microangiopathy have severely impaired anorectal function [6].

The aim of this study was to evaluate the prevalence of enteropathy symptoms and to evaluate anorectal function using high-resolution anorectal manometry in all diabetic patients and patients with select enteropathic symptoms (stool incontinence, constipation, and diarrhea) and duration of diabetes.

## 2. Patients and Methods

The study included 70 individuals: 50 diabetic patients (26 men and 24 women) and 20 non-diabetic (12 men and 8 women) control volunteers. The exclusion criteria for all diabetic patients were as follows: history of neurological conditions (stroke, demyelinating diseases, advanced degenerative disease of the spine), history of spine surgery, an age over 85 years, fasting blood glucose level over 200 mg/dl, and women where the perineal childbirth related injury was greater than the first-degree. The exclusion criteria for the control subjects were: defecation disorders, history of surgical treatment in the minor pelvis and anorectum region, anorectal symptoms, drug intake that could have an impact on the anorectal function, benign prostatic hypertrophy, and women where the perineal injury during childbirth was greater than the first-degree. Control subjects were not diagnosed with any chronic disease. The type of anti- diabetic medications used by patients is illustrated in Table 1 and Table 2. Diabetic patients were divided into subgroups depending on the duration of the disease (less than 10 and over 10 years), symptoms of diabetic autonomic neuropathy (DAN) reported in the questionnaire (with or without DAN symptoms), and glycemic control (based on the reported level of the HbA1c result from the last three months). The diabetic patients were hospitalized in the Department of Angiology, Hypertension and Diabetology, and the Department of Gastroenterology and Hepatology at the University Hospital in Wroclaw. All patients completed the questionnaire, which consisted of demographic and clinical data. Prior to the examination, a test for glucose concentration in capillary blood was collected. Then a digital rectal examination was performed. The evening before the procedure, a preparation with an enema was performed. On the morning of the study patients were fasting and off any medications. The patients underwent the exam in the left lateral position. The anorectal manometry was performed with the ManoScan 360 Sierra Scientific Instruments System. After a digital examination was performed to confirm the absence of stool in the rectum, a high-resolution anorectal manometric catheter with terminal balloon was introduced into the rectum. The examination was performed according to the Rao et al. protocol [7]. The following manometric parameters were evaluated: the maximal anal resting pressure (MRP), the maximal anal squeeze pressure (MSP), cough reflex, the push/strain maneuver, rectal sensation test, the recto-anal inhibitory reflex (RAIR). In addition to the parameters mentioned above, the following were also evaluated: length of the high-pressure zone (HPZ), duration of sustained squeeze, ano-rectal pressure gradient, residual anal pressure (RAP), rectal pressure during the push maneuver, and the rectal compliance. MRP/RAP ratio was calculated.

All patients provided written informed consent to participate in the study, and the study protocol was approved by the human ethics review board of Medical University in Wroclaw (KB-180/2013).

### Statistical Analysis

Numerical data are presented as mean ± standard deviation, median, minimal, and maximal value. The differences between percentages of certain characteristic in each group were determined by chi-squared test. For the differences between two groups, U Mann-Whitney test was used. *p*-value < 0.05 was considered statistically significant.

## 3. Results

At least one chronic symptom was reported by 39 diabetic patients (78%). Symptoms from the lower part of the gastrointestinal tract were reported by 36 (72%) of patients. In Table 3, the frequency of gastrointestinal symptoms in diabetic patients with gender distribution is shown. The most often reported symptom was abdominal bloating (64%). With a similar frequency of patients reporting abdominal pain and a sensation of incomplete evacuation (60% and 62%, respectively). The most often reported symptom from the lower gastrointestinal tract was incontinence of the stool and/or gas and the sensation of urgent defecation (*N* = 22; 44% and *N* = 21; 42%, respectively). Incontinence with liquid stool was reported by 13 patients (26%). The least reported symptom was alternating diarrhea and constipation (*N* = 6; 12%). Chronic constipation and diarrhea were diagnosed with the same frequency (*N* = 15; 30% each group). Anal region discomfort was present in 14 patients (28%). Some symptoms were more often reported by women: incontinence of stool and/or gas (*p* = 0.011), anal region discomfort (*p* = 0.039), as well as a sensation of incomplete evacuation (*p* = 0.016).

Diabetic patients with a disease duration of over 10 years reported anal region discomfort 37% (*p* = 0.028) and a sensation of incomplete evacuation 40% (*p* = 0.036) more often than patients with a shorter duration of diabetes. There were 31 patients with DAN symptoms. Table 4 illustrates the frequency of diabetic autonomic neuropathy symptoms reported by patients in the questionnaire. Patients with symptoms of autonomic neuropathy more often reported abdominal pain 74% (*p* = 0.009) and a sensation of incomplete evacuation 74% (*p* = 0.023) than patients without symptoms. Glycemic control did not influence the frequency of symptoms (Table 5).

Manometric parameters and selected functional tests for all diabetic patients are shown in Table 6 and Table 7. Overall diabetic patients had a lower MSP (*p* = 0.001) and a higher mean threshold of minimal rectal sensation (*p* < 0.01) than the control subjects. In the group of diabetic patients, 8 patients failed to exhibit RAIR, whereas none of the controls did. Evaluation of the push/strain maneuver revealed that diabetic patients had a lower recto-anal pressure differential (*p* = 0.012) and a higher residual anal pressure (RAP) (*p* = 0.044). The differences in MRP/RAP between the two groups were statistically significant and were compatible with the other parameters used to assess the push/strain maneuver.

Evaluation of anorectal function in patients with enteropathy symptoms revealed many differences, which are presented in Table 8 and Table 9. MRP and MSP were significantly lower in all groups of diabetic patients with selected enteropathy symptoms than in the control group *p* < 0.01; *p* < 0.01; *p* = 0.003, respectively, for incontinence, constipation, and diarrhea for MSP and *p* = 0.003; *p* = 0.036; *p* = 0.046, respectively, for incontinence, constipation, and diarrhea for MRP.

Patients with diabetes and chronic constipation had a lower anorectal pressure gradient and MRP/RAP compared to the control subjects *p* < 0.03 and *p* < 0.04, respectively. Whereas patients with chronic diarrhea had only MRP/RAP lower *p* < 0.04 in all the parameters used to evaluate the push/strain maneuver. Significant differences were found in the first sensation threshold between the symptomatic patients and the control group for incontinence, constipation, and diarrhea (*p* < 0.01; *p* < 0.01; *p* < 0.02 respectively). Patients with diabetes and chronic constipation had a higher mean threshold sense of urge to defecate than control subjects *p* < 0.05. Diabetic patients with incontinence and diabetic patients with chronic diarrhea had lower volumes of RAIR first time detection than the control subjects (*p* < 0.01; *p* < 0.04, respectively). RAIR was statistically more significant and frequently absent in the group of diabetic patients with incontinence than in the control subjects (*p* < 0.003).

Evaluation of HRAM variables depending on the duration of diabetes revealed that MRP and MSP was significantly lower in the group of long-standing diabetes (*p* = 0.024; *p* = 0.026, respectively) than in the group of shorter diabetes duration (less than 10 years) (Table 10). The MRP and MSP values of patients with shorter duration of diabetes were comparable to the results of the control subjects.

## 4. Discussion

Seventy-eight percent of diabetic patients reported chronic symptoms from the gastrointestinal tract, which was shown in previous studies [8,9,10]. Symptoms from the lower gastrointestinal tract were reported by 72% of patients. Higher frequency of symptoms from the lower gastrointestinal tract were also observed by Jeong Hawn et al. [10]. The sensation of incomplete defecation was present in 62% of patients, which is more often than in previous studies, 54.3% [11] and 40.5% [9]. The correlation of incomplete evacuation sensation in diabetes was also proved by the study of Jorge et al. [11] and Ihana-Sugiyama et al. [12]. In Jorge et al.’s study, patients with severe incomplete defecation sensation had lower resting anal pressure. In the presented study this symptom was significantly more often observed in the group of long-standing diabetes with the symptoms of DAN. Anal region discomfort was observed in 28% of patients and was significantly more often reported in women and patients with long-standing diabetes. The symptom was more often reported than in the results presented in a single study by Jeong Hawn et al. [10]. The presented correlation with long standing-diabetes is a new observation and needs further research. It can be related to polyneuropathy and DAN, which are more often observed in long-standing diabetes [6,13]. Considering that pudendal nerve fibers innervate the anal canal wall and the skin in the anal region [14], anal region discomfort could be additional evidence of polyneuropathy. Bytzer et al. [15] and Jung-Hwan O et al. [9] in their studies presented equal symptom rates in men and women with no correlation between gender and symptom frequency, respectively. In the presented study, anal region discomfort, sensation of incomplete evacuation, and incontinence were significantly more often reported by women. Pintor et al. presented the correlation between the incontinence and neuropathy [16]. In population-based studies, the appearance of incontinence was not related to gender [17]. In women, except in cases of mechanical injury to the pelvic floor muscles, neuropathy of the pudendal nerves could be observed after childbirth, and could lead to incontinence even many years after labor. Therefore in the authors opinion, diabetes can also be an independent additional risk factor for incontinence in women. Symptoms of diabetic enteropathy such as constipation, diarrhea, and incontinence of the stool and/or gas were reported in 30%, 30%, and 44% of patients, respectively. Twenty-six percent of patients presented incontinence of liquid stool, Epanomeritakis et al. [13] reported frequency of 31.5%, whereas Deen et al. 40% [18] of stool incontinence, which corresponded to the presented results. According to numerous authors, constipation is the most common symptom of enteropathy, present in up to 60% of cases [19,20,21]. The mechanism of constipation is complex, including slow bowel transit and/or dyssynergic defecation. Correlation between the slow transit and the presence of neuropathy was proven [22,23]. Slow bowel transit was also observed among patients with diabetes in the Hye-Kyung J et al. study [24]. ICC (Interstitial cell of Cajal) acts as the neurotransmitters between the enteric nervous system (ENS) and the smooth muscles. The lower amount of ICC and ENS cells in diabetic patients, probably leads to the disturbance in motor function of the gastrointestinal tract including bowels [2,3,25,26,27]. Additionally, myopathy is another etiological factor for enteropathy [3]. The glycemic control (based on the level of the HbA1c) did not influence the frequency of symptoms from the lower gastrointestinal tract, which corresponded with the majority of previous reports. In contrast, Khoshbaten et al. presented a correlation between unbalanced diabetes and higher frequency of constipation and diarrhea (it was based on the questionnaire rather than the HbA1c laboratory results) [28]. There are many positive data considering the correlation between higher HbA1c values and higher frequency of upper gastrointestinal tract symptoms [10,15,29,30,31]. Why a similar correlation is not observed in accordance to the symptoms of lower gastrointestinal tract still needs to be studied. Zhang et al. is presenting the theory about the adverse effect of hyperglycemia on the vagal nerve which leads to the delaying of gastric emptying or other mechanism via the central nervous system [32].

Evaluation of the anorectal function with the use of high-resolution anorectal manometry revealed that squeeze activity of the anal sphincters was impaired in diabetic patients in comparison to the control subjects. The MSP was lower in all diabetic patients, whereas the MRP and MSP were lower in all patients with enteropathy symptoms and patients with long-standing diabetes, as was the MSP/MRP ratio. The results corresponded to some others studies in which both MRP and MSP were lower in diabetic patients [6,13]. In contrast, the results of Deen et al. reported no significant differences in pressure within the anal canal [18], even in the studied group there were patients with incontinence who exhibited incorrect RAIR reflex, suggesting the possibility of neuropathy. This proved the complexity of pathophysiological processes in diabetic complications. Epanomeritakis et al. concluded that there is an increased incidence of incontinence in patients with long standing diabetes [13] (in the presented study, the frequency was also higher, but the result was not statistically significant). This corresponds with the observation of lower squeeze pressure and dysfunction of EAS (external anal sphincter). Lower squeeze activity in the anal canal may be related to motor peripheral neuropathy of pudendal nerves and S3-S4 nerves roots (EAS and pubo-rectalis muscle innervation) [14,33], which is not common in the early stages of diabetes [34] or neuropathy of autonomic nerves, which is responsible for internal anal sphincter (IAS) innervations. The subclinical autonomic neuropathy may appear early in the first year after diagnosis of diabetes type 2 or two years after type 1, whereas the first symptoms may be detected many years later [1]. It was demonstrated that there are special Cajal cells (ICC-M) in IAS in monkeys, which can act as independent starters, not only as transmitters of nervous impulse, as previously thought [35]. The number of ICC cells in diabetic patients with gastrointestinal symptoms decreases, which can be one of the reasons for lower IAS tone [25]. An additional mechanism can be myopathy [2,3] or other mechanisms that are still being studied (as the role of the micro-RNA and its influence on IAS tone) [36]. All diabetic patients had impaired visceral sensation manifested by higher threshold value of first sensation and higher threshold value of urge to defecate noted only in patients with chronic constipation. These results are consistent with previous reports presenting higher threshold values for rectal sensations [6,37]. Patients with longstanding diabetes had much higher values of first sensation threshold than patients with a short history of diabetes but the result was not statistically significant. Probably, the dysfunction of the autonomic regulation of rectum sensation results in hyposensitivity of the rectum. However, Epanomeritakis et al. presented lower threshold values in diabetic patients [13]. Rogers et al. presented no differences according to the visceral sensation test between diabetic patients with peripheral neuropathy, healthy subjects, and patients with idiopathic incontinence [38]. Studies mentioned above present a divergence between the results of the nature of impaired visceral sensation in diabetic patients. The study of Softelan et al. provided evidence of the pathological changes in both autonomic and peripheral nervous systems in diabetic patients [4]. The rectum has autonomic innervations from sympathetic and parasympathetic fibers. Rectal sensation is mediated by parasympathetic fibers that pass from S2 to S4 and impaired rectal sensation in diabetic patients could be additional evidence of DAN [39]. In patients with diabetes and liquid stool incontinence and/or diarrhea, the lower volume of balloon distention was needed for IAS relaxation (RAIR reflex). The absence of RAIR reflex was more often noted in patients with incontinence. Deen et al. also presented impaired RAIR reflex in patients with incontinence [18]. Probably, the observed impaired RAIR reflex is the result of many complex mechanisms such as neuropathy of the rectal nervous plexus, dysfunction of nitrergic neurons in the rectal wall [18], and reduced production of nitric-oxide, a neurotransmitter responsible for smooth muscles relaxation [25,40]. One of the parameters used for evaluation of dyssynergic defecation is the recto-anal pressure gradient. In the presented study, this gradient was significantly lower in all diabetic patients and significantly lower in patients with constipation than in control subjects, which presented enhanced features of dyssynergic defecation and suggest its appearance more often in diabetic patients. Maleki et al., in a small study (only 10 patients) evaluating the dyssynergic defecation in patients with constipation, reported that it was more often observed in patients with constipation and diabetes [41]. These results are in agreement with the presented study and need further research.

### Recommendations for Clinical Practice

The most important issue for the control of the symptoms in the lower gastrointestinal tract in diabetic patients is blood glucose level control. All diabetic patients should be advised lifestyle changes such as extensive physical activity (aerobic and resistance training) at least twice a week, 1000 steps extra a day. It can improve insulin action, glycemic control, lipid levels, and blood pressure. A proper diet is also an important issue [42]. All patients with a slow bowel transit mechanism of constipation should have adequate water and soluble fiber intake (20–30 g per day) [21,43]. Patients with diagnosed constipation related to the dyssynergic defecation are candidates for biofeedback therapy. Following the Rome IV Diagnostic Criteria for Irritable Bowel Syndrome (IBS), methyl cellulose therapy is recommended in chronic constipation. Next to consider are osmotic laxatives which can also be used for patients with dyssynergic defecation. In this group, stimulant laxatives are not recommended as they can worsen the symptoms. Considering impairing bowel movements in diabetes patients using prokinetic drugs such as cisapride can be used as first line therapy [21,43]. Mosapride was effective for severe constipation cases in DM patients both in terms of bowel movement improvement and diabetic control [44]. The diarrhea treatment in DM patients is mostly conservative. Again it should be highlighted that good diabetic control is the most important issue. A diet with low fiber intake and frequent small meal portions are important. Loperamide therapy delays bowel movements and increases the tension of the internal anal sphincter which can be useful also for patients with stool incontinence (2–4 mg 30 min. before the meal—max 16 mg/24 h). In cases of sever diabetic diarrhea, somatostatin analogue therapy can be considered [43,45]. In stool incontinence cases, the alternative treatment can be amitriptyline, which can also reduce urgency to defecate [43,46]. Patients with diabetic stool incontinence, without mechanical injury of the sphincters, can be treated with biofeedback therapy [47]. There are also more advanced methods of treatment (transrectal sphincter stimulation, surgical methods) that can be considered [46].

## 5. Conclusions

In conclusion, symptoms of the lower gastrointestinal tract are often reported by diabetic patients as there is a correlation with impaired anorectal function. In the author’s opinion, diabetes can be an independent and additional risk factor for incontinence in women. Patients with long-standing diabetes presented with lower resting and squeeze anal tone and presented in clinical settings with symptoms such as anal region discomfort and incomplete evacuation sensation. All diabetic patients presented enhanced features of dyssynergic defecation. These symptoms can negatively impact an individual’s quality of life and need to be taken into consideration in diabetic care. The highlight of this study is displaying the relationship between the duration of diabetes and its impact on anorectal function and related symptoms. More advanced supervision and close cooperation between diabetics and gastroenterology specialists should be implemented to improve patient care.

## Figures and Tables

**Table 1:** Type of anti-diabetic medications.

Diabetes Medications	Patients	%
Insulin	12	24
Diabetic oral agents	22	44
Mix treatment (both oral agents and insulin)	16	32

**Table 2:** Diabetic oral agents.

Agent	Patients	%
Metformin	31	62
Sulfonylureas	19	38
Acarbose	3	6
Incretin-based therapy	6	12
Thiazolidinedione therapy	1	2

**Table 3:** The frequency of gastrointestinal symptoms in diabetic patients with gender distribution.

Variables		Sex				DM Patients (*N* = 50)		*p*
		Men (*N* = 26)		Women (*N* = 24)				
		*N*	%	*N*	%	*N*	%	
Abdominal pain	not present	12	46.2%	8	33.3%	20	40.0%	0.355
	present	14	53.8%	16	66.7%	30	60.0%	
Abdominal bloating	not present	8	30.8%	10	41.7%	18	36.0%	0.423
	present	18	69.2%	14	58.3%	32	64.0%	
Anal region discomfort	not present	22	84.6%	14	58.3%	36	72.0%	0.039
	present	4	15.4%	10	41.7%	14	28.0%	
Diarrhea	not present	19	73.1%	16	66.7%	35	70.0%	0.621
	present	7	26.9%	8	33.3%	15	30.0%	
Constipation	not present	21	80.8%	14	58.3%	35	70.0%	0.084
	present	5	19.2%	10	41.7%	15	30.0%	
Alternating diarrhea and constipation	not present	23	88.5%	21	87.5%	44	88.0%	0.917
	present	3	11.5%	3	12.5%	6	12.0%	
Sensation of incomplete evacuation	not present	14	53.8%	5	20.8%	19	38.0%	0.016
	present	12	46.2%	19	79.2%	31	62.0%	
Urgent defecation	not present	16	61.5%	13	54.2%	29	58.0%	0.598
	present	10	38.5%	11	45.8%	21	42.0%	
Incontinence of the stool and/or gas	not present	19	73.1%	9	37.5%	28	56.0%	0.011
	present	7	26.9%	15	62.5%	22	44.0%	

**Table 4:** Diabetic autonomic neuropathy symptoms.

Symptom	Patients	%
Faint	7	14
Tachycardia at rest	16	32
Lower libido, erection dysfunction	17	65
Increased sweating	20	40
Urinary problems	15	30
The lack of typical hypoglycemia symptoms	11	22

**Table 5:** The frequency of gastrointestinal symptoms in diabetic patients depending on glycemic control.

Variables		Glycemic Control				*p*
		Good (*n* = 25)		Bad (*n* = 25)		
		*n*	%	*n*	%	
Abdominal pain	not present	9	36.0%	11	44.0%	0.564
	present	16	64.0%	14	56.0%	
Abdominal bloating	not present	10	40.0%	8	32.0%	0.556
	present	15	60.0%	17	68.0%	
Anal region discomfort	not present	16	64.0%	20	80.0%	0.208
	present	9	36.0%	5	20.0%	
Diarrhea	not present	19	76.0%	16	64.0%	0.355
	present	6	24.0%	9	36.0%	
Constipation	not present	16	64.0%	19	76.0%	0.355
	present	9	36.0%	6	24.0%	
Alternating diarrhea and constipation	not present	21	84.0%	23	92.0%	0.384
	present	4	16.0%	2	8.0%	
Incontinence of the stool	not present	17	68.0%	20	80.0%	0.333
	present	8	32.0%	5	20.0%	
Sensation of incomplete evacuation	not present	10	40.0%	9	36.0%	0.771
	present	15	60.0%	16	64.0%	
Urgent defecation	not present	14	56.0%	15	60.0%	0.774
	present	11	44.0%	10	40.0%	
Incontinence of gas	not present	15	60.0%	16	64.0%	0.771
	present	10	40.0%	9	36.0%	

Good glycemic control for DM type 1—HgA1C ≤ 6.5 and for DM type 2—HgA1C ≤ 7.

**Table 6:** Manometric parameters in diabetic patients and controls.

Variables	DM (*N* = 50)					Control (*N* = 20)					U Mann-Whitney Test
	X	SD	M	Min	Max	X	SD	M	Min	Max	*p*
MRP (mmHg)	84.87	24.39	84.85	18.00	123.90	94.07	12.96	95.50	70.00	115.00	0.133
MSP (mmHg)	164.88	71.95	158.25	20.10	388.10	230.04	59.79	245.15	137.60	326.30	0.001
MSP/MRP	1.96	0.74	1.87	0.92	4.44	2.49	0.79	2.51	1.48	4.66	0.008
HPZ (cm)	3.56	0.77	3.70	1.60	4.80	3.44	0.64	3.50	1.80	4.30	0.362
Duration of sustained squeeze (s)	14.82	5.62	16.80	2.50	20.10	15.07	4.60	16.20	4.00	20.10	0.814
RAP (mmHg)	75.25	27.80	72.10	23.40	133.10	61.38	22.73	52.70	33.30	121.00	0.044
Recto-anal pressure differential	−26.58	29.91	−22.95	−99.40	60.70	−5.52	29.56	−0.90	−62.90	40.80	0.012
The rectal pressure during the push maneuver (mmHg)	47.51	26.46	49.60	−18.50	96.40	55.90	26.46	60.45	11.40	109.20	0.210
RAIR (mL)	44.60	37.10	40.00	0.00	170.00	52.50	14.10	50.00	30.00	70.00	0.053
First sensation (mL)	47.00	25.50	40.00	20.00	140.00	28.50	3.66	30.00	20.00	30.00	<0.01
Defecation threshold (mL)	85.80	38.23	70.00	30.00	200.00	74.00	9.40	75.00	60.00	90.00	0.578
Minimum compliance	−0.11	1.80	0.28	−11.10	0.67	0.37	0.27	0.33	0.13	1.39	0.292
Maximum compliance	4.18	3.54	3.47	1.44	25.60	4.31	4.40	2.93	1.35	19.50	0.304
MRP/RAP	1.22	0.47	1.13	0.54	3.04	1.69	0.56	1.76	0.95	3.06	0.00

MRP—maximal resting pressure; MSP—maximal squeeze pressure; HPZ—high-pressure zone; RAP—residual anal pressure; RAIR—recto-anal inhibitory reflex; X—mean; SD—standard deviation; M—median Min—minimal; Max—maximal; *p* < 0.05.

**Table 7:** Functional manometric tests in diabetic patients and controls.

Variables		DM		Control		*p*
		*N*	%	*N*	%	
RAIR	present	8	16.0%	0	0.0%	0.057
	not present	42	84.0%	20	100.0%	
Cough reflex	correct	48	96.0%	20	100.0%	0.364
	incorrect	2	4.0%	0	0.0%	
Maximum tolerated volume (MTV)	<100 mL	5	10.0%	0	0.0%	0.056
	100–200 mL	10	20.0%	9	45.0%	
	>200 mL	35	70.0%	11	55.0%	

**Table 8:** Manometric parameters in diabetic patients with selected enteropathy symptom.

Variables	Incontinence (*N* = 13)					Constipation (*N* = 15)					Diarrhea (*N* = 15)					Control (*N* = 20)				
	X	SD	M	Min	Max	X	SD	M	Min	Max	X	SD	M	Min	Max	X	SD	M	Min	Max
MRP (mmHg)	69.03	2.15	79c4	18.00	99.00	81.89	17.69	82.90	44.50	115.70	78.14	26.49	80.00	18.00	118.90	94.07	12.96	95.50	70.00	115.00
MSP (mmHg)	120.50	72.57	104.3	20.10	299.7	140.6	47.84	132.3	65.40	229.80	148.96	77.10	143.60	20.10	299.70	230.04	59.79	245.2	137.60	326.30
MSP/MRP	1.79	0.85	1.59	1.02	3.50	1.73	0.49	1.77	0.92	2.67	1.91	0.95	1.62	1.02	4.44	2.49	0.79	2.51	1.48	4.66
RAP (mmHg)	56.32	16.85	58.50	27.30	78.90	71.35	30.70	68.10	23.40	133.10	65.09	26.90	59.10	27.30	121.70	61.38	22.73	52.70	33.30	121.00
RAPD	−9.24	19.3	−12.6	−48.6	21.7	−30.6	26.7	−22.9	−74.4	4.3	−20.6	29.0	−14.7	−84.8	21.7	−5.52	29.6	−0.90	−62.9	40.8
RPPM (mmHg)	47	22.2	50.0	12.8	90.9	40.70	29.10	45.70	−18.5	85.30	44.32	23.86	36.90	12.80	90.90	55.90	26.46	60.45	11.40	109.20
RAIR (mL)	31.54	30.23	30.00	0.00	80.00	51.33	40.68	50.00	0.00	130.00	36.67	30.39	40.00	0.00	100.00	52.50	14.10	50.00	30.00	70.00
FS (mL)	50.00	29.72	40.00	20.00	140.0	58.67	37.20	50.00	20.00	140.00	38.00	13.20	40.00	20.00	60.00	28.50	3.66	30.00	20.00	30.00
DF (mL)	76.92	38.81	70.00	40.00	200.0	106.0	47.48	90.00	50.00	200.00	66.67	16.33	70	30.00	100	74	9.40	75.0	60.0	90.0
MRP/RAP	1.25	0.49	1.22	0.54	2.06	1.35	0.65	1.14	0.60	3.04	1.28	0.46	1.16	0.66	2.06	1.69	0.56	1.76	0.95	3.06

X—mean; SD—standard deviation. M—median Min—minimal. Max—maximal; *p* < 0.05; RAPD—recto-anal pressure differential; RPPM—the rectal pressure during the push maneuver; FS—first sensation; DF—defecation threshold.

**Table 9:** U Mann—Whitney test results for diabetic enteropathy symptoms vs. control subjects.

Variables	Incontinence vs. Control	Constipation vs. Control	Diarrhea vs. Control
	*p*	*p*	*p*
MRP (mmHg)	0.003	0.036	0.046
MSP (mmHg)	<0.01	<0.01	0.003
MSP/MRP	0.017	0.003	0.011
RAP (mmHg)	0.543	0.264	0.764
Recto-anal pressure differential	0.619	0.023	0.177
The rectal pressure during the push maneuver (mmHg)	0.311	0.099	0.167
RAIR (mL)	<0.01	0.489	0.030
First sensation (mL)	<0.01	<0.01	0.015
Defecation threshold (mL)	0.265	0.041	0.102
MRP/RAP	0.037	0.039	0.034

**Table 10:** Manometric parameters in patients depending on the duration of diabetes.

Variables	Diabetes Duration										U Manna-Whitney Test
	<10 Years (*N* = 15)					≥10 Years (*N* = 35)					
	X	SD	M	Min	Max	X	SD	M	Min	Max	*p*
MRP (mmHg)	96.85	22.77	98.00	49.00	123.90	79.73	23.52	82.90	18.00	122.70	0.024
MSP (mmHg)	203.31	79.82	207.90	104.90	388.10	148.41	62.51	151.20	20.10	299.70	0.026
HPZ (cm)	3.65	0.83	3.70	2.00	4.80	3.52	0.76	3.70	1.60	4.60	0.603
Duration of sustained squeeze (s)	13.91	5.71	13.50	2.90	20.10	15.21	5.62	18.20	2.50	20.10	0.482
RAP (mmHg)	81.70	28.35	80.30	38.90	124.90	72.49	27.51	69.20	23.40	133.10	0.295
Recto-anal pressure differential	−27.81	38.01	−22.70	−99.40	60.70	−26.05	26.33	−23.00	−74.40	21.70	0.695
The rectal pressure during the push maneuver (mmHg)	50.03	29.35	53.20	7.50	96.40	46.43	25.51	49.20	−18.50	90.90	0.767
RAIR (mL)	47.33	44.31	40.00	0.00	170.00	43.43	34.21	40.00	0.00	130.00	0.907
First sensation (mL)	38.00	15.21	40.00	20.00	70.00	50.86	28.11	40.00	20.00	140.00	0.120
Defecation threshold (mL)	82.67	40.96	80.00	30.00	200.00	87.14	37.54	70.00	40.00	200.00	0.691
Minimal compliance	−0.35	1.46	0.28	−4.88	0.40	0.00	1.93	0.29	−11.10	0.67	0.058
Maximum compliance	4.18	1.87	3.91	1.61	8.36	4.18	4.07	3.04	1.44	25.60	0.305
MRP/RAP	1.29	0.46	0.13	0.71	2.47	1.19	0.48	1.12	0.54	3.04	0.459

X—mean; SD—standard deviation. M—median Min—minimal. Max—maximal; *p* < 0.05.

## Data Availability

The data presented in this study are not publicly available but are available from the corresponding author on reasonable request.

## Abbreviations

RAP	residual anal pressure
DAN	diabetic autonomic neuropathy
EAS	external anal sphincter
HPZ	high pressure zone
HRAM	high resolution anorectal manometry
IAS	internal anal sphincter
ICC	Interstitial Cajal cells
MRP	maximal resting pressure
MSP	maximal squeeze pressure
RAIR	recto-anal inhibitor reflex
